# Food allergy in a child with de novo *KAT6A* mutation

**DOI:** 10.1186/s13601-017-0155-x

**Published:** 2017-06-22

**Authors:** Varpu Elenius, Tuire Lähdesmäki, Marja Hietala, Tuomas Jartti

**Affiliations:** 1 Department of Pediatrics, Turku University Hospital, Kiinamyllynkatu 4-8, 20520 Turku, Finland; 2 Department of Pediatric Neurology, Turku University Hospital, Kiinamyllynkatu 4-8, 20520 Turku, Finland; 3 Department of Genetics, Turku University Hospital, Kiinamyllynkatu 4-8, 20520 Turku, Finland

**Keywords:** Feeding problems, Food allergy, Histone acetylation, *KAT6A* mutation

## Abstract

Crying combined with miscellaneous gastrointestinal symptoms are typical symptoms of infant with food allergy, but are also common among children with abnormal neurological development. Mutations in *KAT6A* gene is known to cause a syndrome characterized by developmental delay, hypotonia, cardiac defects, microcephaly, specific facial features and early feeding problems. However, these feeding problems have not earlier been specified. We present the first reported case of a DBPCFC confirmed food allergy in a child with *KAT6A* mutation whose feeding problems resolved with elimination diet. The present case does not establish proof of cause, but highlights the importance of careful clinical diagnostics despite other possible causes for feeding problems. Recognizing that early feeding problems these patients regularly have might be caused by food allergy is important for outcome and quality of life for these patients.

## To the editor

Adverse reactions to food are a major clinical problem. The dermatologic and systemic reactions to food are well recognized but reactions manifesting primarily in the digestive tract can be difficult to recognize, diagnose and treat [1]. Most gastrointestinal symptoms are non-IgE mediated [1]. The skin prick test or specific IgE alone are not helpful. Double blind, placebo-controlled food challenge (DBPCFC) is the gold standard for diagnosing food allergy in these patients.

Crying combined with miscellaneous gastrointestinal symptoms are typical symptoms of infant with food allergy. Crying, vomiting and stomach pain combined by feeding problems are also common symptoms among children with abnormal neurological development. These symptoms may be due to child’s muscle hypotonia, oral dyspraxia, aerophagia and obstipation. We present a case to outline the importance of careful clinical diagnostics in case of infant food allergy, despite of other possible causes for feeding problems.

## Case study

**Fig. 1:**
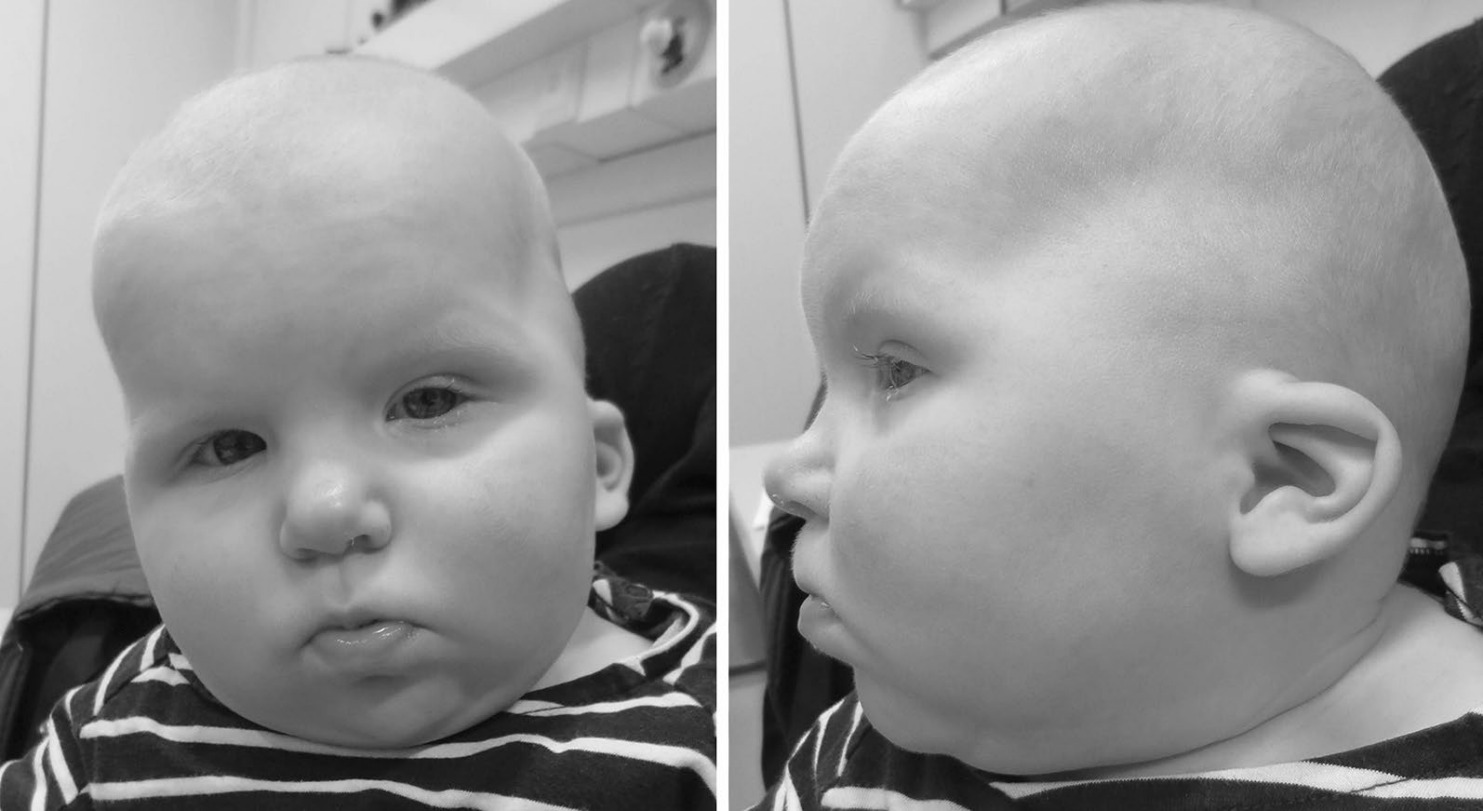
Facial characteristics of the child with *KAT6A* mutation. Characteristic features of this syndrome, e.g. bitemoporal narrowing, broad nasal tip, low set ears, thin upper lip, micrognatia and smooth filtrum are seen

The patient was born at 39 weeks to non-consanquineous parents after uncomplicated pregnancy with normal maternal serum screening and ultrasounds. At birth, weight was 3.0 kg normal, length 46 cm (Z = −2.4) and occipital-frontal circumference (OFC) 33.5 cm (Z = −1.0). At the age of 2 months, she was admitted to pediatric hospital due to poor eye contact. The ophthalmologic examination showed only intermittent esothropia. Further clinical examination showed dysmorphic features (microcephaly, thin upper lip, broad nasal bridge, large forehead, low set ears, epicanthal folds) (Fig. 1), global developmental delay and asymptomatic atrial septal defect and patent ductus arteriosus. Chronic ear infections required tympanic tube placement at age of 20 months and atrial septal defect was repaired at age of 23 months. The achievement of motor milestones was markedly delayed; sitting without support was achieved at 16 months and supported standing at 2 years. No verbal communication was achieved at 2 years. Rehabilitation with physical and speech therapy was initiated during her first year. Brain 3T-magnetic resonance imaging and metabolic screening were normal.

The child was breast-fed until 12 months of age, but additional standard formula was given from 4 months of age. Feeding problems originated at the age of 1 month while recieving only breast milk. Mother noticed that child developed gastrointestinal symptoms (vomiting and diarrhea) if mother had consumed milk or wheat. Symptoms got worse after solids were introduced to diet at age of 6 months. After milk ingestion child had vomiting and diarrhea. Wheat ingestion caused vomiting, discomfort and obstipation. All symptoms resolved with elimination diet. At age of 15 months specific-IgE for milk, wheat, soy, peanut and cod were negative. Therefore, DBPCFC with milk and wheat was performed. DBPCFC test confirmed wheat and milk protein allergy. The child had vomiting, discomfort and obstipation during double blind wheat challenge test, and vomiting and diarrhea during double blind milk challenge test. With the elimination of wheat and milk protein her feeding problems and gastrointestinal symptoms resolved. At 2 of years old, her OFC was less than third percentile for age but weight and length increased appropriately with elimination diet.

The patient was referred to clinical geneticist. Many of the features exhibited in this child exist in some well-characterized genetic syndromes like Down, Rett and genitopatellar syndromes, but genetic testing for these syndromes were normal. In order to understand the molecular mechanisms underlying the phenotype of this child, DNA was isolated from peripheral blood by standard procedures and whole exome sequencing (WES) was performed [2]. Parents provided written informed consent for WES, and parents own DNA was isolated for comparison. DNA samples from the family were sent to whole exome sequencing at Centogene laboratory, Rostock, Germany, a commercial laboratory offering WES as a diagnostic service. First, exons were enriched from fragmented genomic DNA and then the sample was processed on the NextSeq Platform (Illumina, San Diego, California, USA). A previously unreported heterozygous variant was detected in exon 17 of the *KAT6A* gene, c.3655del (p.Leu1219Tyrfs*). The identified variant was confirmed by Sanger sequencing. This deletion creates a frame shift and the new reading frame ends in a stop codon 74 positions downstream. This variant was absent in the parents.

## Discussion

*KAT6A*, also known as *MOZ* or *MYST3*, is a lysine (K) acetyltransferase that is located on chromosome 8p11.2. It was first found as a fusion protein in patients with acute myelogenous leukemia [3]. In humans, *KAT6A* is expressed in 49 different tissues including brain, heart and intestine. The protein forms a part of a histone acetyltransferase complex that acetylates lysine-9 residues in histone H3 (H3K9). Acetylated H3K9 is associated with transcriptionally active genes, whereas histone deacetylation is associated with gene silencing. Thus, *KAT6A* has important roles in gene-specific histone 3 acetylation, regulating developmental gene expression and controlling a wide range of processes such as cell mobility, metabolism, DNA replication and repair.

A year ago, mutations in *KAT6A* was shown to cause a syndrome characterized by developmental delay, hypotonia, early feeding problems, cardiac defects, microcephaly and specific facial features such as broad nasal tip, bitemporal narrowing, thin upper lip, low set ears [4, 5]. All of these features were found in our patient (Fig. 1). Previous studies have also reported non-specific feeding problems. Arbodel et al. [4] reported that 3/4 patients had feeding problems and 2/4 had reflux. In the study of Tham et al. [5] 5/7 had feeding problems requiring nasogastric tube or gastrostomy. We contacted the authors of these reports, but in many cases these patients were never tested for food allergies. In Arbodel’s study group at least 2/4 patients had been later diagnosed with food allergy. The existence of food allergy in these patients may be coincidental and further research is needed to confirm this possible link. Biased immune events can be expected since *KAT6A* has frequently been associated with a failure to maintain a normal number of haematopoietic precursors suggesting a specific role of *KAT6A*-driven acetylation in controlling a critical balance between proliferation and differentiation in haematopoietic progenitor and stem cells [6].

To our knowledge, this is a first report showing a DBPCFC confirmed food allergy-related gastrointestinal symptoms in a child with *KAT6A* mutation whose feeding problems resolved with elimination diet. The present case does not establish proof of cause, but highlights the importance of careful clinical diagnostics despite other possible causes for feeding problems. Recognizing that early feeding problems these patients regularly have might be caused by food allergy is important for outcome and quality of life for these patients.

## Abbreviations

DBPCFC: double blind placebo-controlled food challenge
IgE: immunoglobulin E
WES: whole exome sequencing
